# Uncoupling the Effects of Seed Predation and Seed Dispersal by Granivorous Ants on Plant Population Dynamics

**DOI:** 10.1371/journal.pone.0042869

**Published:** 2012-08-07

**Authors:** Xavier Arnan, Roberto Molowny-Horas, Anselm Rodrigo, Javier Retana

**Affiliations:** 1 CREAF (Centre de Recerca Ecològica i Aplicacions Forestals), Facultat de Biociències, Universitat Autònoma de Barcelona, Bellaterra, Catalonia, Spain; 2 Unitat d'Ecologia, Departament de Biologia Animal, Biologia Vegetal i Ecologia, Facultat de Biociències, Universitat Autònoma de Barcelona, Bellaterra, Catalonia, Spain; University of Utah, United States of America

## Abstract

Secondary seed dispersal is an important plant-animal interaction, which is central to understanding plant population and community dynamics. Very little information is still available on the effects of dispersal on plant demography and, particularly, for ant-seed dispersal interactions. As many other interactions, seed dispersal by animals involves costs (seed predation) and benefits (seed dispersal), the balance of which determines the outcome of the interaction. Separate quantification of each of them is essential in order to understand the effects of this interaction. To address this issue, we have successfully separated and analyzed the costs and benefits of seed dispersal by seed-harvesting ants on the plant population dynamics of three shrub species with different traits. To that aim a stochastic, spatially-explicit individually-based simulation model has been implemented based on actual data sets. The results from our simulation model agree with theoretical models of plant response dependent on seed dispersal, for one plant species, and ant-mediated seed predation, for another one. In these cases, model predictions were close to the observed values at field. Nonetheless, these ecological processes did not affect in anyway a third species, for which the model predictions were far from the observed values. This indicates that the balance between costs and benefits associated to secondary seed dispersal is clearly related to specific traits. This study is one of the first works that analyze tradeoffs of secondary seed dispersal on plant population dynamics, by disentangling the effects of related costs and benefits. We suggest analyzing the effects of interactions on population dynamics as opposed to merely analyzing the partners and their interaction strength.

## Introduction

Seed dispersal is one of the most ecologically significant plant-animal mutualisms [1], [2], and it is central for understanding plant population and community structure and dynamics [2], [3]. As with many other interspecific interactions [4], [5], seed dispersal by animals also involves both costs and benefits [2]. Their balance determines whether the net outcome falls between mutualism or antagonism [2], [6]. Potential benefits of secondary seed dispersal (i.e., a multistep process with two or more phases, which involve different dispersers that usually extend the distance from the seed's parent plant) are mainly the colonization of new areas, the reduction of parent-offspring competition and of density-dependent predation, as well as the possible dispersal towards microsites that are favorable for establishment [7]. However, secondary seed dispersal is usually preceded by seed removal, which may reduce recruitment through seed consumption [2], [8]. Throughout this study we use the term ‘seed dispersal’ and ‘seed predation’ to refer to the benefits and costs of secondary seed dispersal, respectively. The impact exerted by dispersers may vary from dispersing all seeds intact to destroying nearly all seeds [2], [9]. The net outcome might be context-dependent [1], [2], [5], [10] and may depend on the composition of the assemblage, environmental conditions and fruiting neighborhoods [2], but also on diaspore traits and crop sizes [2], [11], [12].

Although there is a great deal of literature on seed dispersal, very little information is still available on the effects of dispersal on plant population dynamics [13]–[15]. Even less is known about the tradeoffs of secondary seed dispersal. There is thus a need for examining seed dispersal interactions within the context of plant population dynamics [2], [16], [17]. Understanding the combined effect of benefits and costs of dispersal is not straightforward. Experimental studies, in which one side of the interaction is eliminated while the other is controlled for [18] are often difficult to carry out, because removing one side of the interaction would almost unavoidably affect the other one. A useful approach then is to use simulation models which allow analyze the two ecological processes separately. Theoretical or data-based simulation models allow the researcher to test hypotheses regarding ecological processes that would otherwise be too complicated or cumbersome to test experimentally. A simulation model is therefore a convenient tool for integrating the two sides of an interaction and thus obtaining accurate short- and medium-term predictions of overall dynamics in either actual (i.e. observed distributions of species number and sizes) or simulated scenarios [19]–[21].

Many ant-plant interactions show ecological tradeoffs (i.e., a combination of benefits and disadvantages derived from the interaction) [22], and costs of seed dispersal by ants are among the most important ones [1], [2], [23]. Ants may play an important role in the dynamics of plant communities by acting either as seed dispersal agents or as seed predators, or both [24]–[26], [27], [28]. There are two main mechanisms through which ants disperse seeds. One is myrmecochory, or seed dispersal mediated by the elaiosome, i.e., a lipid-rich seed appendage that mainly attracts non-granivorous ants and provides rewards for seed dispersal [1], [28], [29]. The other one is diszoochory, or seed dispersal performed by seed-harvesting ants [8], [24], [25], [27], [30] that is not mediated by any particular seed structure. While the former has traditionally been recognized mainly as a mutualism [1], [28], [29], the latter is usually perceived as an antagonism [31], [32]. Although there are many experimental studies of the effects of seed predation and seed dispersal by ants on plant population dynamics [25], [31], [32], it is still unclear to what extent a population's current state depends on their relative balance. For this reason it is crucial to distinguish the effects related to each of these ecological processes and to analyze their implications in the dynamics of plant populations. It has been recently stated that the net outcome of ant-plant mutualisms is relatively consistent, being beneficial on average [22]. However, this is difficult to assume in seed dispersal systems involving seed-harvesting ants, where the patterns of seed predation and seed dispersal are highly dependent on seed attributes and primary seed dispersal mechanisms [25], [30].

Here we explore the issue of how to disentangle seed dispersal and seed predation associated to secondary seed dispersal by seed harvesting ants on plant population dynamics through a data-based simulation approach. This framework is applied to the dual role of seed-harvesting ants on the populations of plant species characterized by different plant attributes, mainly seed size and their corresponding primary dispersal mechanism, in a heterogeneous environment. For this purpose, we implement a spatially-explicit, individually-based stochastic simulation model based on field data that determined the dynamics of three shrub populations on the short and medium term. Our previous works in the same study area showed that these plant species strongly interact with seed-harvesting ants that prey on and disperse their seeds [25], [26], [30], thus suggesting that ants might condition plant population dynamics through seed dispersal and seed predation. These species also account for different plant attributes, particularly seed size and primary dispersal mechanism. Experimental data collected at field included the initial plant abundance and distribution, seed production, primary seed dispersal, seed removal rates, seed drop in trails and seed rejection at refuse piles by seed-harvesting ants, as well as seed germination, seedling and adult survival rates for each plant species. The model enabled us to compare four different scenarios that arise from the combination of the two ecological processes (i.e., seed dispersal and seed predation; see below) and thus makes it possible to analyze separately their effects on plant populations. We hypothesize that the effects of seed predation and seed dispersal may depend on plant attributes, hence allowing us to define theoretical plant models in response to the dual role of harvesting ants.

## Materials and Methods

### Ethics Statement

All necessary permits were obtained for the described field studies. Our study was conducted with the permission of the land owner where the study area was located. Field studies did not involve endangered or protected species. All experiments comply with current Spanish and international laws.

### Study system

#### The study area

The data were collected in an open and heterogeneous shrubland located in Castellbell i el Vilar, Barcelona (northeast Spain, 41°39′N, 1°51′E), at 260 m above sea level, where the climate is typically Mediterranean, with a mean annual temperature of 14.5°C and a mean annual precipitation of 565 mm. The study area is described in detail in [30]. The vegetation type was the consequence of recurring fires in a *Pinus halepensis* forest. *P. halepensis* recovery was absent after the last fire in 2003. The study was conducted between 2005 and 2010, so vegetation was still at a very early stage in the secondary succession. In order to describe the heterogeneity of the plant cover in the study area, we defined four microhabitats with increasing plant density: (i) bare soil, with none or very few plants; (ii) low sparse vegetation, with individuals of different plant species under 40 cm high surrounded by areas without vegetation cover; (iii) low dense vegetation, similar to the former but without bare areas and dominated by young individuals of different woody species; and (iv) high vegetation, with herbaceous and woody plants over 40 cm high. These microhabitats showed differences in several environmental variables such as solar radiation, temperature and dry weight of herbaceous vegetation (see [25], [30]).

#### The seed-harvesting ant guild

The seed-harvesting ant guild of the study areas was composed of three species belonging to the *Messor* genus: *M. barbarus*, *M. bouvieri* and *M. capitatus*. These species have a broad Mediterranean distribution, mainly in open, sunny environments typical of western Mediterranean areas [33], [34]. They may have an important role in the plant population dynamics [25], [30] and plant community composition [35], [36] of these environments through seed predation and seed dispersal processes. The three species are active all year round [37], so they were obviously active during the primary fruit dispersal stage. For a detailed description of the foraging behavior of these three ant species, see [26].

#### Natural history of the three shrubby species

*Coronilla minima* L. (Fabaceae) is a woody plant species <45 cm high that lives in dry, open shrublands. The fruit is a legume with two to five elongated seeds (mean±SE seed weight 10.0±0.6 mg, seed dimensions 1.4±0.0×10.8±0.6 mm). The seeds disperse by gravity either individually or in groups of a few seeds when the fruit breaks open. Flowering and fruiting periods occur from May to August [38]. One-year-old individuals of this species are already mature enough to produce seeds; thus the span of its reproductive cycle is considered to be 1 year. *Fumana ericoides* (Cav.) Gandg. (Cistaceae) is a woody species <40-cm high that is frequently found in calcareous, dry shrublands. The fruit is an ovoid capsule with 8–12 seeds (mean 2.2±0.1 mg; 1.2±0.0×1.8±0.0 mm). Seeds disperse by gravity after fruit dehiscence. Flowering and fruiting are bimodal, with a first period from February to July, and a less important second period from September to October [38]. In the study area, we observed flowers and fruits in most 1-year-old individuals; thus 1 year is the time considered to be the span of a reproductive cycle. *Dorycnium pentaphyllum* Scop. (Fabaceae) is a woody species 20 to 150 cm high that is commonly found in Mediterranean grasslands and shrublands. The fruit is an ovoid capsule containing one to two rounded seeds (weight 3.2±0.1 mg; dimensions 1.4±0.0×1.7±0.0 mm). These seeds disperse by explosion of the ballistic fruits. The flowering and fruiting periods last from April to August [38]. Its reproductive cycle spans 2 years, and 1-year-old plants are not yet reproductive.

#### The initial scenario

In an area of ca 1,800 m^2^ we established a network of squared cells with a mean area of 0.25 m^2^ (0.5×0.5 m) each (for a total number of 7,066 cells, which represents complete coverage of this area). Each cell was characterized by its dominant (i.e. larger apparent cover area) microhabitat type, by the presence or absence of adults of the three plant species and by the existence of nests of any *Messor* species. This sampling was carried out in late June 2005 and marked the starting point of our study system. The study area was initially composed of high (37%), low sparse (35%) and low dense (17%) vegetation cells, as well as bare soil (11%) cells. *F. ericoides* was the species most present in the area (48% of cells), followed by *C. minima* (29%), whereas *D. pentaphyllum* (13%) accounted for a low representation. *Fumana ericoides* and *C. minima* plants were mainly associated with microhabitats of low sparse vegetation (44% and 41% of cells, respectively), while *D. pentaphyllum* occupied high vegetation cells (46%). There were 27 colonies of the three *Messor* species within the study area (15, 7 and 5 of *M. barbarus*, *M. capitatus* and *M. bouvieri*, respectively). The characteristics (i.e., vegetation type, aspect, slope) of the study area were very similar to the surroundings and to the entire burned area.

### The model

A stochastic, spatially explicit simulation model was implemented to uncouple the effects of two opposed and closely linked ecological processes, i.e. seed predation and ant-mediated seed dispersal on the dynamics of the three plant populations in a heterogeneous environment. The algorithm was written in Visual Basic© 6.0 and the output consisted of Miramon GIS [39] maps showing the squared cells occupied by each seed and plant in the study field. The simulation worked by following the life-history of each individual seed, from production to dispersal (abiotic or biotic), and then eventual establishment and growth as an adult plant, or to disappearance. Ants acted as predators and seed dispersers. Seeds that were picked up by ants could, once lost on the way to the nest and subsequently forgotten by the ant, establish and grow at exactly the same rates as unremoved seeds. Following [24], we assumed that ants did not damage or harm the seeds when transporting them. The seeds that were not lost could then reach the nest and either be eaten (which took them out of the simulation definitively) or stored on a pile of rejected seeds (yet still able to germinate and grow) by the nest entrance.

We modeled all the ecological processes involved in all plant life cycle stages with the help of data collected in field observations and experiments, as shown in the subsequent sections. Prior to the computations, and given that the shorter (vertical) side of the study area was less than twice the maximum secondary dispersal, we expanded the original area to try to lessen or minimize border effects. It was decided that the new expanded range, which included the original area plus a buffer zone, would not receive any influx of seeds, nor would it be subjected to any visit by ants from areas outside its borders, hence isolating the new area from any external input. Moreover, any seed whose randomly-determined abiotic dispersal distance might take it out of the expanded area would instead see its dispersal recalculated again until it fell within the area borders. In other words, the seeds could only move within the new area. Seed disappearance could therefore occur only through ant predation.

A model run consisted of a simulation in which the algorithm progressed from year 0 to year 5. The model was only applied to a 5-year period since it is in the early years after disturbance that changes in plant populations of Mediterranean shrublands are highest. This approach enabled us to properly evaluate the effects of these two antagonistic ecological processes. Morever, we had obtained data with which to validate the model five years after the beginning of the study. The initial scenario for each model run was started by randomly populating cells in the study area with adult plants. Cells with the presence of at least one plant in the 2005 census were labeled as “occupied”. The number of plants per cell was then calculated by drawing numbers from the observed distribution of abundance per plant species (see the Model parameterization section). Plants from two or three different species could occupy the same cell independently, since there was no interaction between plant species in the model at any stage. Ultimately, this made the simulation equivalent to three different simulations, one for each plant species. Initial cell microhabitat types and ant nests were chosen to match those in the 2005 observations. Although microhabitat types could change randomly from one year to the next, ant nest numbers and spatial locations were assumed to be the same through all the simulations because their renewal rate was low.

**Figure 1:**
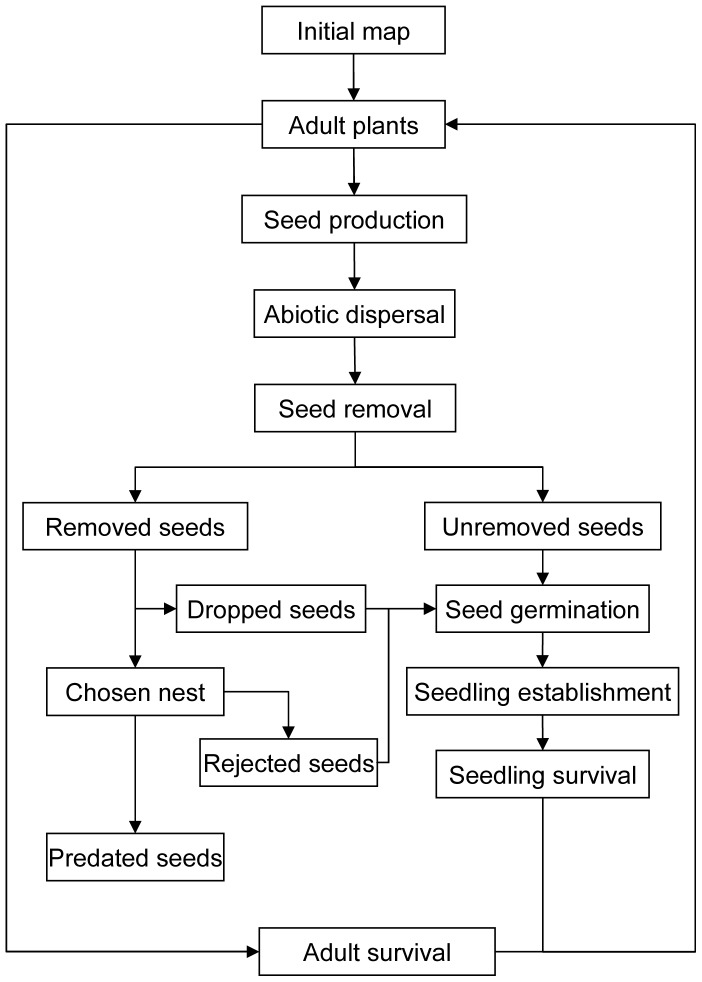
Simple control flow diagram of the simulation algorithm. Ecological processes that have been incorporated into the algorithm to specify the life cycle of a plant species, including the interaction with secondary seed dispersers. After an initial map (year 0) of plant number per occupied cell is determined, the simulation starts by letting adult plants (top of the diagram) produce seeds which may then follow different paths through the algorithm. They may end up either as predated seeds (which are simply eliminated from the model) or as unremoved, dropped or rejected seeds, all of which may germinate and, eventually, give rise to adult plants again. In the diagram these processes are equivalent to going from top to bottom and, then, back up to the top again. That represents one time step (i.e. one year) in the model, and one whole model run consists of simulating a time period of five years. The simulation follows the fate of every plant and seed individually, both in time (yearly) and space (in each cell).

After setting up the initial scenario, the algorithm worked by evaluating a concatenation of ecological processes at each step that included, in the following order: seed production, abiotic dispersal, seed removal, seed drop or nest arrival, seed rejection/consumption, adult mortality and, finally, seedling germination and establishment and adult survival. Some of these processes (i.e., germination, predation, adult mortality, seed germination and mortality) were calculated as binomial processes for individual seeds or plants. The computation of these ecological processes in the model is explained in detail in the sections below. A schematic diagram of the algorithm can be seen in Figure 1. Only cells belonging to the original study area, not to the larger, expanded one, were saved on output. Cells belonging to the original study area were tagged and followed separately. Temporary results at each annual step were saved as GIS maps for subsequent analysis. Temporal dynamics of output values could thus be studied per plant species. As mentioned above, the implementation of these processes in our simulation model is such that plants, seeds and seedlings are tracked individually. In that respect, the algorithm is that of a spatially explicit, individual-based model. Other methodologies, like e.g. matrix population models, are less suited for tracking the characteristics of single elements through time and were discarded early in the study.

Several assumptions were made during model development and implementation: a) Plants, seeds and ant nests were always assumed to be placed at cell centers, and dispersal could only take place between cell centers; b) Microhabitat types changed randomly between types at each time step, following transition rates which had been observed in the field (Table S1); c) Seeds were more likely to be picked by ants from nearby ant colonies; d) A seed lost by an ant could not be picked up again by another ant, effectively giving that seed a chance to germinate; e) No distinction was made between the different ant species.

#### The scenarios

The model was run under four different scenarios which arose from the cross combination of the two antagonistic effects, i.e. seed predation and ant-mediated seed dispersal. The scenarios were as follows: i) The ‘dual effect’ scenario, where ants may predate and disperse seeds; this corresponds to a full model; ii) The ‘disperser’ scenario, where ants disperse but do not predate seeds. In this case, all seeds removed by ants but not dispersed (i.e. seeds that reach the nest and are not rejected on the refuse piles) are relocated again at the end of each simulation step (i.e. year) to the cells from which they were previously removed; iii) The ‘predator’ scenario, where seeds are only predated and not dispersed by ants. In this case, all seeds that are removed and should be dispersed under the full model are relocated again at the end of each simulation step (i.e. year) to the cells from which they were previously removed; and iv) The ‘no-ant’ scenario, where no seed predation and seed dispersal by ants take place, and then the seed removal stage is removed from the full model. This scenario is the only one without ants.

**Figure 2:**
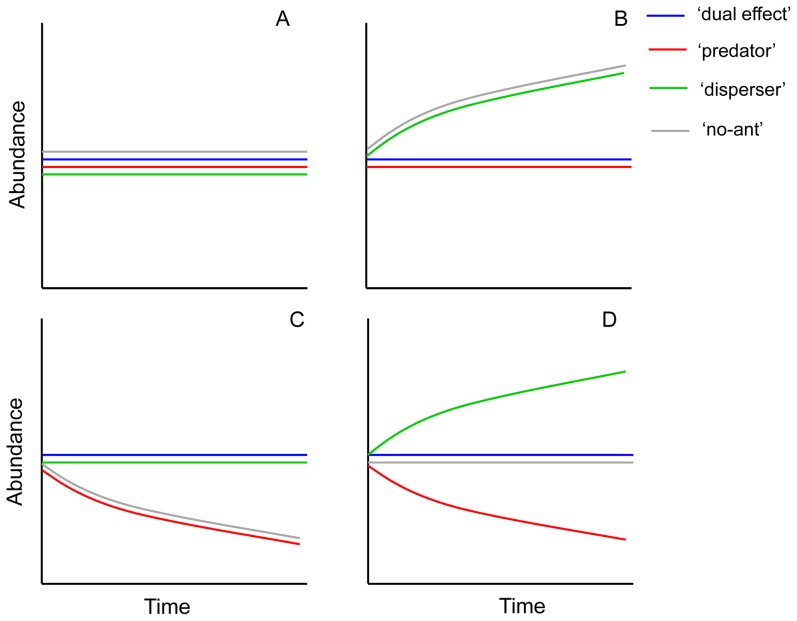
Theoretical models of plant population dynamics responses to the dual effect of seed harvesting ants. Graphical representation of the main four different theoretical models of plant population dynamics (A, Ants-independent model; B, Ant predation-dependent model; C, Ant dispersal-dependent model; and D, Ant-dependent model) in response to the uncoupled effects of seed predation and seed dispersal by ants which are determined by the comparison of four different scenarios in which each is the result of the cross combination of the two antagonistic effects (‘dual effect’ scenario, Predation+Dispersal+; ‘predator’ scenario, Predation+Dispersal−; ‘disperser’ scenario, Predation−Dispersal+; and ‘no-ant’ scenario, Predation−Dispersal−). Minus and plus represent the absence or existence of an effect, respectively. Note that the “dual effect” scenario was used as the reference scenario and, thus, it was settled with constant values thorough time and models.

An examination of the plant population dynamics generated by the simulation model in these scenarios prompted us to consider four general models of the response of plants to the dual role of ants (Figure 2). These models can be described as follows: a) An “ant-independent model”, where the plant population dynamics from the four different scenarios follow the same pattern. Any effect of ants on the corresponding species through seed predation and seed dispersal is either nil or much lower than that of any other, unknown factor (Figure 2A); b) An “ant predation-dependent model”, where the plant population dynamics determined by the ‘predator’ scenario follows the same pattern as the ‘dual effect’ scenario, and the ‘no-ant’ and ‘disperser’ scenarios follow a dynamic above the ‘dual effect’ scenario (Figure 2B). In this model, plant population dynamics clearly rely on the detrimental effects of seed predation by ants and this interaction corresponds to a typical antagonism in which ants benefit from seed predation at the expense of plants; c) An “ant dispersal-dependent model”, where the pattern of the ‘disperser’ scenario parallels that of the ‘dual effect’ scenario, and the patterns of both ‘predator’ and ‘no-ant’ scenarios display lower values (Figure 2C). In this model, plant population dynamics are positively determined by ant seed dispersal effects, and this interaction corresponds to a typical mutualism in which ants and plants derive a mutual benefit; and finally d) An “ant-dependent model”, where plant population dynamics from the ‘no-ant’ scenario parallel the ‘dual effect’ scenario, and the ‘disperser’ and ‘predator’ scenarios have values that are higher and lower, respectively, of the ‘dual effect’ scenario, (Figure 2D). This model shows that plant population dynamics rely on both seed predation and seed dispersal by ants, and the state of the plant population depends on the outcome of the strength of each effect.

#### Outputs of the model

The simulation model was run for 5 years in order to evaluate the progression of the colonization/extinction pattern of each plant species in relation to its interaction with seed-harvesting ants. A total of 100 model runs were randomly performed for each of the four scenarios. The outputs from the model were: a) Spatial occupation, computed as the relative number of cells occupied by at least one individual; b) Plant density, computed as the mean number of adult individuals per cell; c) Pair correlation function, 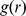, which was measured between cells with plant presence, and which is defined as:


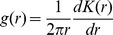


Where *r* is distance and 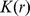 is the well-known Ripley's *K* function [40]; d) Occupancy rate, computed as the relative number of cells with the presence of at least one individual with respect to the total number of cells that were occupied at the beginning of the study; and e) Colonization rate, computed as the number of occupied cells in relation to the number of cells that were initially empty. All these parameters were computed for each simulation year and for the original study area (i.e. cells in the buffer zone were not counted). Ripley's *K*, which is usually applied to the analysis of point process data [40], has also been shown to be a useful tool to study the characteristics of the spatial patterns in presence-absence lattice data [41]. Furthermore, the pair correlation function 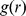 derived from Ripley's *K* is related to the probability of finding an occupied cell at a given distance of another occupied cell; that is, 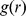 is similar to a probability density function [42]. This makes the interpretation of 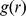 relatively easier than that of 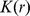 to understand how the distribution of intercell distances changed between species and scenarios. In order to obtain a smooth 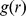 curve, we first calculated 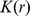 and then averaged it over 100 simulations, for every species, scenario and year combination. The function 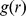 was then evaluated from the resulting mean Ripley's *K*. Both 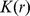 and 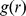 were determined using the R statistical software Version 2.12.1 [43] with the package ‘spatstat’ Version 1.26-1.

To assess the differences among scenarios, we preferred to work in qualitative terms rather than by using statistical inference. This is because our results are based on simulations and the statistical power obtained from the number of available simulations is disproportionate. Thus, differences in the outputs between scenarios were assessed from relative magnitudes between the mean values of two different scenarios using a reference cut-off point of ±10%. To simplify matters, such differences were only evaluated at the fifth year of simulation, except for the pair correlation function, where they were not only evaluated with the values predicted by each scenario in the fifth year of simulation, but also with the real values of the initial scenario.

#### Comparison of model results with observed data

To compare the model predictions with real data, the whole study area was sampled again in late June 2010 (five years after the initial sampling of the study area). We characterized each cell by its microhabitat type and the presence or absence of adults of the three plant species. The proportion of occupied cells could then be calculated, and compared to those predicted by the simulation model under the more realistic ‘dual effect’ scenario.

### Model parameterization

From the information obtained in the field for the different stages of the life cycle of the three plant species (both those including interaction with seed-harvesting ants and those without such interaction), we elaborated different real data sets and functions to parameterize the processes that take place during the different life cycle stages, from seed production to adult survival. With the exception of dispersal, all parameters from other life cycle stages were characterized for each microhabitat type.

#### Plant abundance

The abundance of adult individuals per cell was measured in a subsample of 50 cells for each microhabitat type in 2004. Random numbers of adult individuals were then generated from this distribution of adult abundance to populate cells that had been tagged as “occupied” in the initial study map. This was done per species and per microhabitat type (see Table 1 for mean and standard deviation values).

**Table 1:** Model parameterization.

	*C. minima*	*F. ericoides*	*D. pentaphyllum*
Plants per cell	2.0 (1.5)	4.02 (3.4)	1.9 (1.3)
Seed production	111.2 (237.7)	25.3 (37.8)	165.4 (443.6)
Seed removal	51.2	79.1	79.1
Seed germination	32.5	6.5	8.7
Seedling survival	31.2	20.7	28.9
Adult survival	98.6	86.1	98.3 (91.7)

Observed mean and standard deviation (in parenthesis) of number of plants per spatial cell and seed production per individual plant; rates (in %) of seed removal by ants, seed germination, seedling survival and adult survival per plant species are also shown. Adult survival rate for 1-year *D. pentaphyllum* plants is given in parenthesis.

#### Seed production

Seed output per plant was measured following the methodology of [25] (see also File S1 for specific methodological details). Each value was individually picked at random from its respectively observed distribution. Table 1 shows the mean and standard deviation of seed production per plant species.

#### Primary seed dispersal

The methodology to determine primary seed dispersal is described in thorough detail in [25], [30] and File S1. Here we will only describe how it has been implemented in the model. We assumed a simple exponential distribution function for the dependence of gravity seed dispersal upon distance *x*, such as:


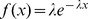


where 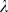 is the inverse of the expected value of the distance *x*. Seed counts were determined at increasing distance intervals from the parental plant. We then integrated over angle and distance interval to obtain the probability for a seed to fall within the distance interval (r, r+10) cm from the plant:


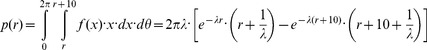


We used the nonlinear regression module in STATISTICA 6.0 to fit the 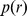 curve to our data. The observed *C. minima* and *F. ericoides* seed proportions were satisfactorily modeled by this curve. For *D. pentaphyllum* data to be well fitted, on the other hand, we had to use the sum of two curves like the one shown above. The observed data and the fitted curves are shown in Figure S1. Once computed, the corresponding cumulative distribution function could be determined from 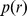. Then, abiotic seed dispersal distances per plant species could be calculated by randomly drawing distance values from their respective dispersal cumulative distribution functions. A computed dispersal distance and direction that took a given seed beyond the borders of the expanded area was recomputed again until it remained within the borders of the area.

#### Seed removal

Seed removal rates by ants were evaluated following the methodology used by [25] (see also File S1). These removal rates were therefore incorporated into the model as a binomial process and are shown in Table 1.

#### Seed drop in trails

To determine seed drops, we assessed the fraction of seeds collected by ants that were then dropped as described in [25], [30] and File S1. The respective distributions of seed drop distances per plant species were fitted with the 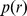 curves explained above. These curves were then used to derive a theoretical, smooth seed-dropping curve up to a distance of 17.25 m, which corresponded to the maximum observed drop distance. The resulting curves and corresponding data are shown in Figure S1. Random biotic dispersal distances were then computed following the procedure already adopted for abiotic dispersal (see above).

Before being picked up by an ant, the algorithm had to choose a nest for a seed to head for. The probability of its being taken in the direction of a given nest was assumed to be proportional to the inverse of the seed-nest distance, so that seeds were more likely to be picked by ants from a nearby nest. Subsequently, when the distance to the nest of choice was shorter than the random drop distance computed from the curve above, the seed was assumed to have disappeared into the nest (although it could be rejected later on; see below). Otherwise, a two-tailed binomial test could not reject, for any of the three plant species, the null hypothesis that the average simulated (*C. minima*: 10.9%, *F. ericoides*: 9.8%; *D. pentaphyllum*: 15.0%) and the field-observed (*C. minima*: 12.2%, *F. ericoides*: 10.0%; *D. pentaphyllum*: 13.7%; see File S1) proportions of dropped seeds were indistinguishable (data from [25], [30], *C. minima*: # successes = 85, # trials = 821, p-value = 0.62; *F. ericoides*: # succ. = 71, #tr. = 708, p-value = 0.80; *D. pentaphyllum*: # succ. = 107, # tr. = 722, p-value = 0.96).

#### Seed rejection at refuse piles

To evaluate the number of seeds transported and deposited on the refuse piles of *Messor* nests, and which were potentially able to germinate, we followed the methodology carried out by [24] (see also File S1). Mean and standard deviation values corresponded to 14.4±44.1 and 39.9±143.8 seeds per nest and year for *F. ericoides* and *C. minima*, respectively. Data for *D. pentaphyllum* are not shown since its corresponding refuse pile was always empty. At each time step in the model we then used that distribution of seed numbers to generate random number of rejected seeds per ant nest. Note that seed rejection is not a percentage from seeds that reach the nest, but an absolute number of seeds according to the observed distribution of seeds deposited on the refuse piles.

#### Seed germination

We measured the germination of all unremoved or dispersed seeds (i.e. on ant trails or refuse piles) as described in [25], [30] and File S1. Mean values for each plant species are shown in Table 1. Germination rates were incorporated into the model as a binomial process.

#### Seedling and adult survival

Seedling (and 1-year old *D. pentaphyllum* plants) and adult survival rates were assessed as described in [25], [30] and File S1. Survival rates per plant species are shown in Table 1. Rates were then incorporated into the model as a binomial process.

#### Random mortality from competition

In addition to seedling and adult survival we implemented two other mortality processes in the algorithm. The goal was to account for competition effects in a more precise way (not directly tested in this study). The first process introduced a threshold or ceiling to the number of plants per spatial cell, and per plant species separately, above which no other plant could establish. That threshold corresponded to 22 plants for *F. ericoides*, 10 for *C. minima* and 6 for *D. pentaphyllum*. These ceiling numbers were calculated from field data as maximum observed plant abundance per cell and species. Simulated values of plant abundance that happened to be higher would subsequently be trimmed down to those values at each time step. This first mortality process effectively prevented some cells in the simulated study from reaching high-density values. The second process limited the rate of occupied (i.e. containing at least one plant) cells that became empty in one time step, as well as the number of empty cells that were occupied by at least one plant after one time step. First, we took field measurements of both rates, per plant species, from 2005 to 2007 and converted the biannual rates to yearly values (Table S2). Next, we assessed in the simulations whether the rate of newly emptied cells (i.e. cells that lost all plants from one time step to the next) between two time steps of the simulation was lower than the one observed, and we randomly emptied all plants of a given number of cells until the two rates matched. Analogously, we checked to see whether the rate of newly occupied cells (i.e. cells that, although empty at one time step, showed at least one established adult plant at the next time step) between two time steps of the simulation was larger than the observed one, and we then randomly emptied as many cells as necessary to make the two rates match. The number of cells to be emptied was first calculated with the full model corresponding to the ‘dual effect’ scenario and then used as such in the particular simulated cases in the ‘disperser’, ‘predator’ and ‘no-ant’ scenarios. This second mortality process effectively prevented the simulated study area from filling up too quickly with occupied cells.

## Results

### Spatial occupation

**Figure 3:**
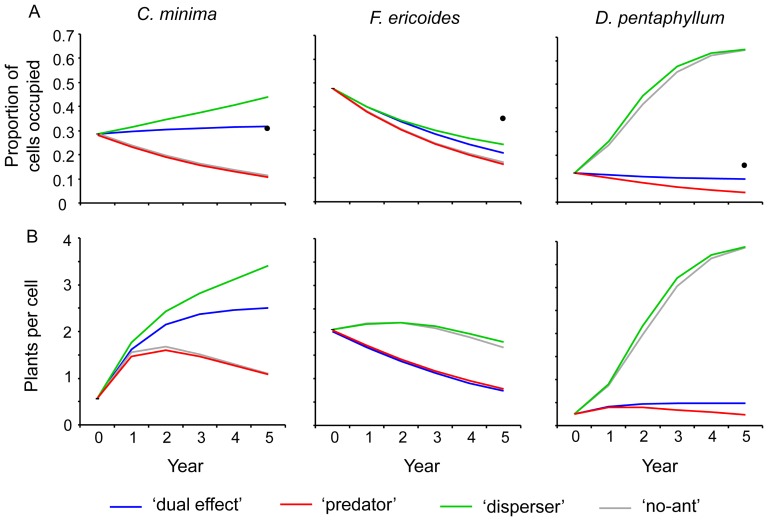
Spatial occupation and plant density predicted by the simulations model run. (A) Proportion of cells occupied, and (B) plant density per cell (0.25 m^2^) of *C. minima*, *F. ericoides* and *D. pentaphyllum* predicted by the simulations model run under the four different scenarios (‘dual effect’, ‘predator’, ‘disperser’, and ‘no-ant’) and for each simulation year. The black circles indicate where the observed occupation values fall five years after the beginning of the study (validation data).

The results from the simulation models showed that cell occupation of *C. minima* steadily decreased with time for the scenarios without ant seed dispersal (Figure 3A). The tendency displayed by the ‘dual effect’ and the ‘disperser’ scenarios was positive and with higher values than those of the other two scenarios (‘no-ant’ and ‘predator’ scenarios). Thus, this species decreased its occupancy if there was no seed dispersal mediated by ants, and consequently it mostly followed an ant dispersal-dependent model. Since the ‘dual effect’ scenario showed lower values than the ‘disperser’ scenario, this would suggest a small negative effect of seed predation. However, this effect is questionable because the values of the ‘predator’ scenario (including only predation, not dispersal) are similar to those of the ‘no-ant’ scenario.

Regarding *F. ericoides*, the pattern of cell occupation for all scenarios followed a pattern of temporal decline that was similar to that of the ‘dual effect’ scenario (Figure 3A). This means that the spatial occupation of *F. ericoides* did not depend on ants, but on other factors. Moreover, although this species seemingly follows an ant-independent model, the pattern of differences among the four scenarios, with both ‘predator’ and ‘no-ant’ scenarios being lower than the other two, is reminiscent of the differences seen for *C. minima*. This suggests a small, though measurable, impact of ant-mediated seed dispersal on the *F. ericoides* occupation pattern.

Finally, the cell occupation dynamics for *D. pentaphyllum* clearly and strongly depended on seed predation by ants, rather than on seed dispersal, since both the ‘disperser’ and the ‘no-ant’ scenarios showed much higher proportion values than the other two scenarios. Our results indicate, therefore, that *D. pentaphyllum* follows the ant-predation dependent model (Figure 3A). There was also a small, though positive effect of seed dispersal, as seen by the fact that the values of the ‘predator’ scenario extended slightly below those of the ‘dual effect’ scenario. However, non-separation of the values from the ‘disperser’ and ‘no-ant’ scenarios (with and without seed dispersal, respectively) raises doubts about this small effect.

### Plant density

The temporal dynamics of plant density (Figure 3B) of *C. minima* followed a pattern similar to that of cell occupancy. Plant density strongly depended on seed dispersal and, to a lesser extent, on a possible effect of seed predation. Consequently, this species also followed an ant dispersal-dependent model. Results for *F. ericoides*, on the other hand, indicated that plant density in general decreased with time, although the scenarios differed in the details. The two curves for the ‘disperser’ and ‘no-ant’ scenarios descended far more gradually than the other two, even showing a slight increase until the second year. This indicated that *F. ericoides* follows an ant predation-dependent model with regard to plant density. With respect to *D. pentaphyllum* plants, this species followed exactly the same pattern developed in the case of cell occupancy, with plant density being highly dependent on seed predation. This fact corroborates that this species follows an ant predation-dependent model.

### Pair correlation functions

We averaged Ripley's *K* over the 100 simulated presence-absence spatial maps for every combination of scenario and species (for an example of plant abundance maps from which presence-absence maps are computed, see Figure S2). The corresponding pair correlation functions *g(r)* were computed from this averaged *K(r)*. The calculations were done for the starting cell distribution and for the resulting simulated distribution after running the model for 5 years. Given that we were only interested in a comparative study of 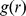 curves between species and scenarios, no border correction to 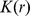 was introduced.

**Figure 4:**
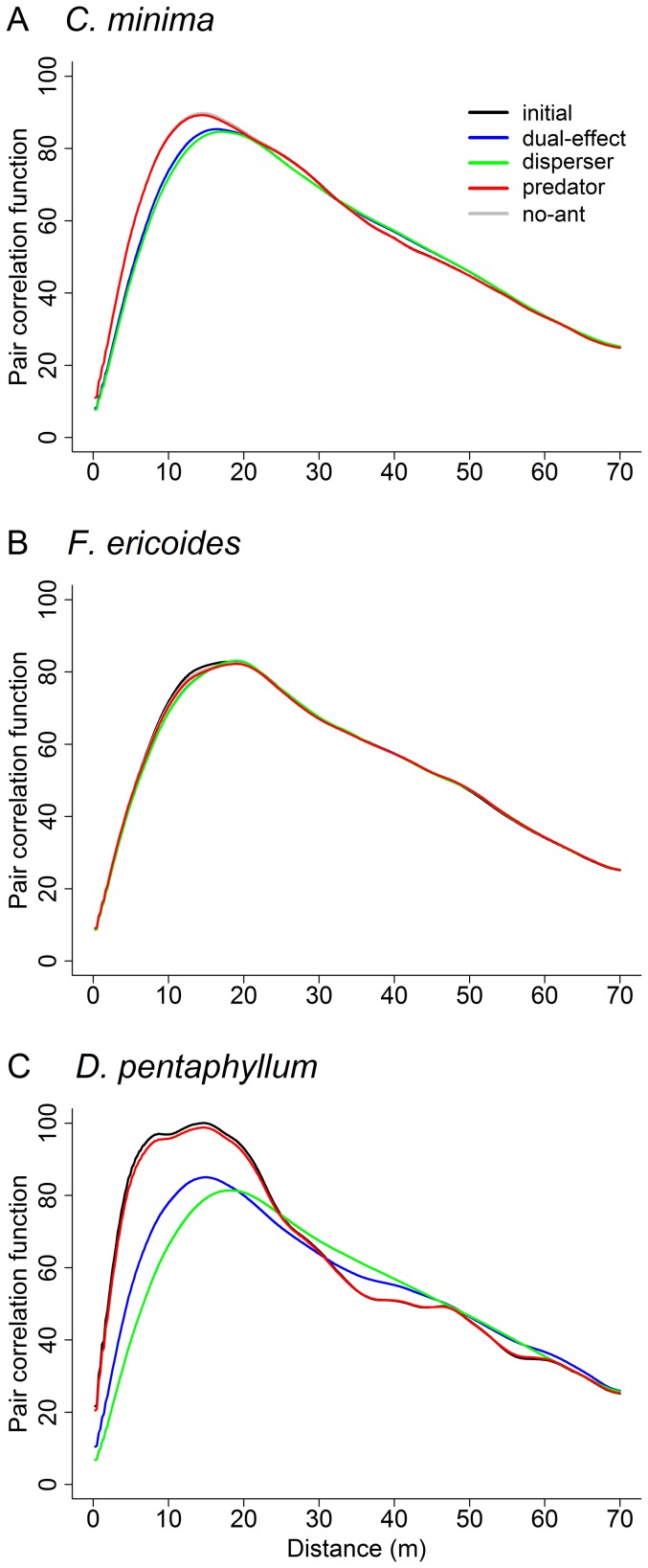
Pair correlation functions. Pair correlation functions 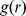 calculated from mean Ripley's 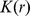 curves (see text for details) from simulated plant presence-absence maps. Shown are results corresponding to *C. minima* (A), *F. ericoides* (B) and *D. pentaphyllum* (C) after the simulation algorithm ran for 5 years. For the sake of comparison, the corresponding 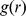 curves for the initial scenario (i.e. the observed spatial distribution of presence-absence of plants for each species) are also included and are labeled as ‘initial’. The gray curve (‘no-ant’) has been drawn but is barely visible in the figures. It is under the red curve (‘predator’) in A and B, and under the green curve (‘disperser’) in C.

All 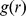 curves (Figure 4) first showed rapid growth, which peaked at about 10–20 m, followed by a long, slowly decreasing tail towards longer distances. The latter effect is caused by the limited extension of the study area and the lack of a border correction in 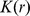, which gives rise to a deficit in points located at increasingly long distances. A comparison between curves at all distances is nevertheless enlightening. A detailed examination of Figure 4 revealed that 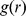
varied between plant species and, noticeably, between scenarios and years. The results for *C. minima* (Figure 4A) after five years under the scenarios with seed dispersal (i.e. ‘dual-effect’ and ‘disperser’) deviated perceptibly from those without seed dispersal (i.e. ‘no-ant’ and ‘predator’) and from the initial scenario. Therefore, seed dispersal increased the proportion of longer distances between pairs of cells with presence of *C. minima* plants. These results point to *C. minima* obeying an ant dispersal dependent model. *Fumana ericoides* 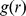 curves (Figure 4B), on the other hand, clearly corresponded to that of an ant independent model, since the distributions of intercell distances were almost identical for all scenarios. That is, *F. ericoides* dynamics seem to be conditioned by external factors, rather than by the presence of seed-harvesting ants. Finally, the dynamics of *D. pentaphyllum*, illustrated by the corresponding 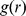 curves in Figure 4C, conformed to that predicted by an ant predation dependent model. Scenarios with seed predation (i.e. ‘dual effect’ and ‘predator’) displayed higher proportion of shorter distances than scenarios without (i.e. ‘no-ant’ and ‘dispersal’). Furthermore, when only seed predation was considered (i.e. ‘predation’ scenario), the corresponding curve and the one for the initial scenario were nearly identical. That is, seed predation did not allow the spatial extension of the *D. pentaphyllum* population by primary dispersal or seed dispersal by ants.

### Occupancy and colonization rates

**Figure 5:**
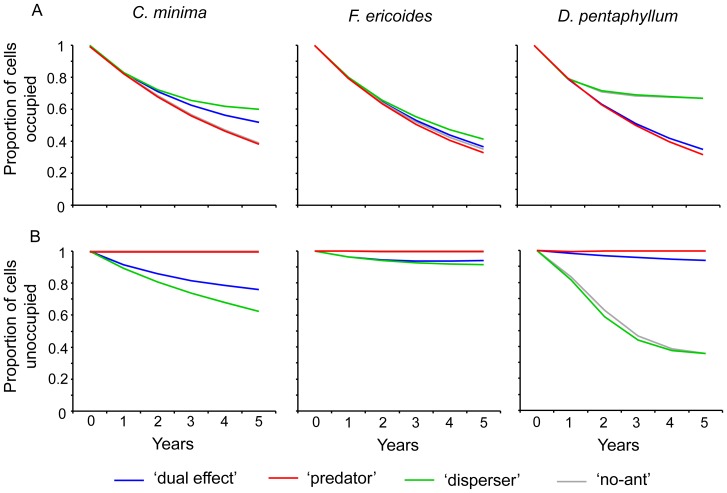
Occupancy and colonization rates calculated from simulations. (A) Proportion of cells occupied each year of simulation in relation to all cells that were occupied in the initial scenario (occupancy rate), and (B) proportion of cells unoccupied each year of simulation in relation to all cells that were unoccupied in the initial scenario (colonization rate) of *C. minima*, *F. ericoides* and *D. pentaphyllum* predicted by the simulations model run under the four different scenarios (‘dual effect’, ‘predator’, ‘disperser’, and ‘no-ant’).

The occupancy rate of the *C. minima* population was higher in scenarios with seed dispersal by ants (i.e. the ‘dual effect’ and ‘disperser’ scenarios) (Figure 5). This fact could be due to the recolonization by seed dispersal mediated by ants from cells where adults had recently disappeared. The results also indicated that occupation of empty cells only took place under the simulation scenarios with ant-mediated seed dispersal (Figure 5). Colonization of new cells never took place without seed dispersal by ants. This result suggests that the colonization rate of *C. minima* is clearly dependent on seed dispersal by ants. This species clearly fits an ant dispersal-dependent model with regard to both the occupancy rate and the local expansion of the population. As regards of *F. ericoides*, the occupancy rate was similar among scenarios (Figure 5), while the colonization rate was very low, and only occurred in scenarios with seed dispersal by ants (similarly to *C. minima*). Consequently *F. ericoides* agrees well with an ant-independent model and an ant dispersal-dependent model when it comes to occupancy and colonization rates, respectively. As for *D. pentaphyllum*, the occupancy rate was higher in models without seed predation (i.e. the ‘no-ant’ and ‘disperser’ scenarios) (Figure 5). This could be related to the likelihood that recolonization of recently unoccupied cells would be easier in these scenarios where predation is not an obstacle. Furthermore, colonization of empty cells took place in the same scenarios without seed predation, and even slight colonization occurred in the ‘dual effect’ scenario, where ants predated but also dispersed seeds because several seeds escaped predation. This species clearly acts as an ant predation-dependent plant species in relation to the occupancy and colonization rate.

### Comparison of model results with observed data

The observed global proportion of cell occupation for *C. minima*, *F. ericoides* and *D. pentaphyllum* in 2010, five years after the initial sampling of the study area, was 29%, 33% and 15%, respectively. The corresponding cell occupation percentages, as predicted by the ‘dual effect’ (i.e. full) simulations, were 32%, 21% and 10%, respectively. The closest agreement between observation and simulation corresponded to *C. minima*, followed by *D. pentaphyllum* and at lesser extent to *F. ericoides* (Figure 3). More interestingly, the values predicted by our simulation models followed the same pattern as the real pattern, with a certain stability of cell occupation in the case of *C. minima* (from 25% of observed occupancy at the start of the study to 29% and 32% of observed and predicted occupancy five years later) and *D. pentaphyllum* (from 13% of observed occupancy at the start of the study to 15% and 10% of observed and predicted occupancy five years later), and with a high decline in *F. ericoides* (from 48% of observed occupancy at the start of the study to 33% and 21% of observed and predicted occupancy five years later).

## Discussion

In this paper we have successfully separated and analyzed the costs and benefits of seed dispersal by seed harvesting ants on plant population dynamics. The study was made possible by implementing a stochastic and spatially explicit simulation model based on real data sets that considers all the life cycle stages of plant regeneration. Our study is arguably an important contribution to the field of plant population dynamics and seed dispersal effectiveness. Firstly, there is a need of studies examining the plant-disperser interaction within the context of plant population dynamics in general [2], [16], [17], and particularly in the case of plant-ant interactions. This necessity is even more pronounced within the context of their costs and benefits [4]. Secondly, it has been argued that our understanding of seed dispersal effectiveness would be greatly improved with more spatially explicit approaches that consider potential conflicts between different stages of the life cycle [2]. By comparing four different scenarios which are the outcome of the cross combination of the two sides of the interaction (seed predation and seed dispersal), our simulation model allowed to analyze which of the two ecological processes (or none) may affect the population dynamics of three shrubby plant species with different biological attributes. The comparison of the four proposed scenarios initially help us establish a series of theoretical models of plant responses to the dual role of ants, depending on the extent to which plant population dynamics depend on seed predation and/or seed dispersal by seed-harvesting ants (Figure 2). Then, the agreement with some of these theoretical models indicates whether ants are important to a particular plant species as dispersers (plants that follow an ant-dispersal dependent model), as predators (plants that follow an ant-predation dispersal model), both (plants that follow an ant-dependent model), or as neither of the two (plants that follow an ant-independent model).

Our results have showed that seed-harvesting ants may have strong effects on plant population dynamics at local scales, and that these effects are mediated by seed predation, but also by seed dispersal. These results confirm previous experimental studies that predicted the dual role of seed-harvesting ants as predators and dispersers [24], [25], [30], even though they had long been regarded only as predators. Likewise, results also showed that the three plant species studied agree with a different theoretical model. Plant responses were thus highly variable, displaying positive, negative, and even no effect on seed dispersal. This conditional outcome might result from species plant attributes. For instance, *C. minima* clearly fits an ant-dispersal dependent model (see Table 2). The temporal pattern of spatial occupation, distribution and density of individuals all depend on ant-mediated dispersal (Figure 3, 4, 5). This is likely the consequence of the limited abiotic dispersal mechanisms of this large-seeded species, which barely enables seeds to move away from the parent plants. The high seed production of this species (Table 1) may be a crucial point for explaining the low impact of ant predation. However, the removal rate (about 50%) should be high enough to finally bring about effective seed dispersal on account of the low seed drop rate (12.2%). Meanwhile, *D. pentaphyllum* is clearly associated with an ant predation-dependent model (Table 2), as can be seen in the results corresponding to the spatial occupation, distribution and density of individuals (Figure 3, 4, 5). A mechanism of abiotic dispersal over long distances allows this species to colonize new cells without the help of ants, which is reflected in a null effect of ants through seed dispersal even though it is the species with the highest seed drop rate (13.7%). Otherwise, and despite its high seed production of small seeds (Table 1), seed predation has negative effects on its temporal dynamics, thus preventing further spatial expansion.

**Table 2:** Summary of the theoretical models of plant population dynamics in response to seed predation and seed dispersal followed for each plant species and output of the model.

Output	*C. minima*	*F. ericoides*	*D. pentaphyllum*
Spatial occupation	Ant dispersal-dependent model	Ant-independent model	Ant predation-dependent model
Plant density	Ant dispersal-dependent model	Ant-predation dependent model	Ant predation-dependent model
Spatial aggregation pattern	Ant dispersal-dependent model	Ant-independent model	Ant predation-dependent model
Stability rate	Ant dispersal-dependent model	Ant-independent model	Ant predation-dependent model
Colonization rate	Ant dispersal-dependent model	Ant dispersal-dependent model	Ant predation-dependent model

Regarding *F. ericoides*, this species mainly follows an ant-independent model (Table 2). Although the seeds of this species also have a high removal rate (about 90%, similar to *D. pentaphyllum* seeds), seed predation only affects the individual density of this species, and there is no predation effect on its spatial occupation and distribution. Moreover, seed dispersal mediated by ants is not important for the dynamics of this species despite displaying seed drop values (10%) that are similar to those of *C. minima*. Like *C. minima*, *F. ericoides* also has a dispersal mechanism limited to very short distances. However, even though ants allow seeds to move beyond the microhabitats of production and colonize new areas, seed dispersal by ants does not lead to effective dispersal. This might be related to the low seed germination rate of this species in relation to *C. minima* (Table 1). However, the results suggest that some unknown factor is driving the population dynamics of this species, more than the action of ants. The scarce adjustment between observed values and those predicted by the model under the ‘dual effect’ scenario (i.e. the only realistic scenario under our study system) supports this assertion. Indeed, the high spatial initial occupation of this species in the area (48%), along with a fairly uniform spatial distribution (Figure 4, Figure S2), may play an important role in this unexpected result according to its biological attributes. In many populations of long-lived perennials, recruitment is limited by the availability of safe sites rather than by seed supply [44], [45]. Consequently, the lack of available empty cells to colonize may mask both seed predation and seed dispersal. This suggests that the net outcome of seed dispersal at local scale might be context-dependent, because it may rely highly on plant population sizes [5], [10]. This directly points to the conditional outcome concept [2], [5], [6], [22], [46], where the outcome of an interaction is context dependent. Our findings are in agreement with other studies that state that, while other kind of interactions are consistently mutualistic or antagonistic across ecological contexts [22], seed dispersal interactions are quite conditional [2], [10]. For instance, the foraging behavior of a particular ant species may determine seed removal, predation and dispersal rates as well as seed dispersal distances (for myrmecochorous ants [47], for seed harvesting ants [25]). Moreover, the rapid change of ant communities along geographic gradients [48], [49], [50] will have a varying impact on plant fitness through seed dispersal and seed predation. Also, it has been reported that the presence of one seed species can modify the seed removal rates by rodents of other seed species [51], which could also be applied to ants.

In this sense, an interaction in which the costs and benefits can simultaneously affect population dynamics in a completely opposite way may not be chimerical. In fact, a recent study that analyzes ant-seed interactions within an evolutionary framework [52] reached similar conclusions. The authors found that seeds of *H. foetidus* that were dispersed by ants were subjected to two contrasting partial selective scenarios, because seed removal and seedling establishment selected for seed size in different directions, limiting the evolutionary potential of seed dispersal. In our study none of the three study plant species paralleled the ants-dependent model, where both costs and benefits are simultaneously at work. An species that fits this model would be one with constrained primary seed dispersal, and therefore, a species that would require ants to colonize new areas. In turn, this colonization should also be limited by a negative effect through seed predation. Consequently, the net balance of the two opposed processes would not cause either positive or negative effects on the plant population dynamics.

Costs and benefits are not always evident from an interaction, and only one of them is often evident [5]. In our case study we addressed an interaction that has been traditionally accepted as an obvious antagonism, where only the associated costs have been highlighted in the past. Nonetheless, when analyzing its effects on plant population dynamics in detail through costs and benefits, we have found that this interaction can also result in a neutralism or even a mutualism. Rather than studying the partners and magnitude of the interaction, there is a particular need to analyze the effects of such interactions on plant population dynamics that will really determine which kind of interaction it is.

This is one of the first works that analyze the effects of secondary seed dispersal on plant population dynamics, which is particularly novel for ant-seed dispersal interactions. We demonstrated that the outcome of seed dispersal interactions is strongly dependent on plant species, driven by their attributes and probably also by the environmental context. In their review about seed dispersal effectiveness, Schupp et al. [2] highlighted a relevant current discussion on the effects of diplochory, i.e., when and where two seed dispersers are better than one. In our study system, costs associated to secondary seed dispersal constraint the abundance of a species that is able to colonize the entire study area by its own primary dispersal mechanism, whereas benefits associated to secondary seed dispersal facilitate the spread on another species across the study area which is not able to do it on its own. Whatever the outcome for a given species, this conditional outcome, which is driven by species attributes, might in turn promote plant species coexistence at a local scale, and consequently promote species diversity. Our spatially explicit and stochastic model provides a framework for forecasting spatial and temporal distribution of individual plants from the initial plant species distribution pattern, and with the help of parameters measured at all regeneration stages. By comparing different scenarios where some regeneration stages are modified or removed, their effects can be brought to light. This type of models opens the door to analyze the effects of a wide range of seed dispersal interactions on plant population dynamics. To this aim, a good knowledge of the biology of the species considered is nevertheless needed. Moreover, the inclusion of specific and precise parameters describing competition relationships among plant species might help to go one step beyond and analyze the effects of secondary seed dispersal, through the associated costs and benefits, on species coexistence at the community level.

## Supporting Information

File S1 Detailed description of the methodology carried out at field to get the data used to parameterize the different life cycle stages of the three plant species that have been implemented in the model.
(DOC)

Figure S1 **Experimental data and fitted curves for primary and secondary seed dispersal, per plant species.** Figures correspond to *C. minima* (*A* and *D*), *F. ericoides* (*B* and *E*) and *D. pentaphyllum* (*C* and *F*). All three figures on the left column show primary dispersal results, whereas those on the right column correspond to seed dispersal by ants through seed drops.
(TIF)

Figure S2 **Initial distribution of plants and example of simulated distribution after 5 years.** Observed distribution of plants for *C. minima* (A), *F. ericoides* (B) and *D. pentaphyllum* (C), and example of an output from the simulation model for the same species (labels D, E and F, respectively). The study area is outlined by a continuous solid line. The diameter of bullets is proportional to plant abundance. An abundance of 10 plants in a cell corresponds to the bullet drawn at the upper right corner of the figure, outside the study area.
(TIFF)

Table S1 Yearly transition matrix (in %) for microhabitat types based on field observations. We first carried out field measurements between 2005 and 2007 and converted those biannual rates to yearly values. Abbreviations: BS, bare soil; LSV, low sparse vegetation; LDV, low dense vegetation; and HV, high vegetation.
(DOC)

Table S2 Rates (in %) of newly emptied and newly occupied cells as employed in the random mortality module of the simulation algorithm, per plant species.
(DOC)
