# Supplementary material for: Uncoupling the Effects of Seed Predation and Seed Dispersal by Granivorous Ants on Plant Population Dynamics
Source: PLoS One. 2012 Aug 7;7(8):e42869. doi: 10.1371/journal.pone.0042869 (PMC3413678; doi:10.1371/journal.pone.0042869)
Supplement: File S1 — Detailed description of the methodology carried out at field to get the data used to parameterize the different life cycle stages of the three plant species that have been implemented in the model. (DOC) [file pone.0042869.s001.doc]

**Supporting Information S1.** **Detailed description of the methodology carried out at field to get the data used to parameterize the different life cycle stages of the three plant species that have been implemented in the model.**

*Seed production*

Seed output per plant was measured in 10 randomly distributed individuals per species and per microhabitat in 2004 [1, 2]. We counted the number of fruits per individual at the peak of maximum production. We estimated mean number of seeds per fruit by counting the number of seeds in 50 fruits, which were randomly collected from a mixture of plants from the four microhabitat types. Therefore, we are implicitly assuming hereafter that the seed:fruit ratio does not depend upon habitat type. Seed production per species was calculated, for every cell, as the product of number of individuals, number of fruits per individual and number of seeds per fruit.

*Primary seed dispersal*

Primary seed dispersal was measured in the field in the period of highest seed production in 2004 [1, 2]. The terrain surrounding a sample of five different plants for each plant species was surrounded by a circular 1.5-radius plastic tray smeared with Tanglefoot® (Tanglefoot Co., Grand Rapids, MI, USA) to catch and secure fallen seeds and avoid seed removal. One month later, the trays were removed and taken back to the laboratory, where seed number was counted in concentric 10 cm-width circular rings.

*Seed removal*

To evaluate rates of seed removal by ants, we placed seeds in transparent plastic tubes 6 cm in length and 0.5 cm in diameter that were fixed to the ground to prevent disturbance by rodents and birds [3, 4]. Previous field observations confirmed that ants readily found seeds of the three study species within these tubes. In each habitat type, we placed four replicates, with 10 tubes each, i.e., 160 seeds per species. This sampling was repeated in two different periods of activity of granivorous ants: summer (July 2005) and autumn (September–October 2004), although data were analysed by pooling together the two sampling periods. Seed removal in each device was monitored 30 days after seed placement in the field [1]. From our own observations we assumed that seeds that had not been removed by ants after those 30 days were very unlikely to be removed in the future.

*Seed movement: seed drops*

To determine seed drops, we assessed the fraction of seeds collected by ants that were dropped (that is, not consumed and abandoned far from the mother plant) and their respective seed drop distances by tracking seed dropping by loaded *Messor* workers returning to the nest. Workers carrying a seed were followed from the moment they collected it (either found naturally in the field or offered by the observer) until they reached the nest or dropped the seed. We measured the distance travelled by the ant while carrying the seed. We carried out about 50 measurements per plant species in 15 different nests (5 nests of each ant species: *M. barbarus*, *M. bouvieri* and *M. capitatus*). Overall, we followed 2,251 seed-carrying ants (821, 722 and 708 carrying *C. minima*, *F. ericoides* and a *D. pentaphyllum* seed, respectively). The measurements were performed throughout the year in 2004 and 2005, with a total observation period of 400 h [1, 2].

*Seed movement: seed refuse piles*

A small proportion of the seeds transported into the *Messor* nests were rejected intact and collected in refuse piles. To evaluate the number of seeds of the three plant species deposited in the refuse piles of *Messor* nests, and that were potentially able to germinate, the entrance of fifteen nests was cleaned up in 2005. Ten days later, we collected the refuse piles produced in these nests and transported them to the laboratory, where we counted the number of seeds and fruits of the three plant species found among the debris. The number of seeds rejected intact and kept in the refuse pile was used as an estimator of the number of seeds that had gone uneaten. This sampling was repeated in different periods of the year (June, August and October) according to the fruiting peak of the three plant species considered. We extrapolated the total number of seeds of each species deposited in the refuse piles by multiplying the values obtained per day by the total number of days of activity of these ant species throughout the year. The distribution of seed numbers was very variable, with a strong peak at zero. Mean and standard deviation values corresponded to 14.4±44.1 for *F. ericoides* and 39.9±143.8 for *C. minima*. Data for *D. pentaphyllum* are not shown since its corresponding refuse pile was always empty.

*Seed germination*

To determine seed germination of all those unremoved or dispersed (i.e. in ant trails or in refuse piles) seeds, we used laterally perforated pots (11 x 11 x 12 cm) buried in the ground, leaving 2 cm over ground level. These pots were filled with substratum belonging to the corresponding microhabitat. In each pot, we deposited 10 seeds per species previously collected in the study area. All pots were covered with a 0.5-cm mesh to avoid predation by vertebrates and by large invertebrates [3]. In addition, the part of the pot above the ground was smeared with Tanglefoot® (Tanglefoot Co., Grand Rapids, MI, USA) to prevent access of small invertebrates, mainly harvesting ants. In each of the four microhabitats, we randomly placed 10 replicates and a control pot without seeds to evaluate eventual germination of seeds already present in the substratum or dispersed from vegetation. As germination in controls was almost absent, no correction was carried out on the observed germination rates. Pots were installed in early October 2004, and seeds were placed in late October 2004. The number of germinated seeds was monitored monthly from November 2004 to June 2005 [1, 2]. The results allowed us to calculate the germination rate per plant species and microhabitat type.

*Seedling survival*

We marked 20 randomly distributed seedlings per species in each microhabitat type (i.e., 80 seedlings per species) in late autumn 2005, just after seed germination and seedling establishment. We monitored these seedlings again 1 year later to evaluate seedling survival [1, 2]. As 1-year-old *D. pentaphyllum* plants were not yet reproductive plants, we also measured the survival rate of this species in the change from 1-year-old plants to adults. Twenty 1-year-old *D. pentaphyllum* plants were marked in each microhabitat in late winter 2006 and monitored again 1 year later.

*Adult survival*

In order to estimate adult survival twenty randomly distributed adult plants per species were tagged in each habitat type (i.e., 80 adult plants) in late-winter 2006 and were monitored again 1 year later [1, 2]. Survival rates were computed per plant species and microhabitat type from the observed data.

REFERENCES

1. Arnan X, Rodrigo A, Molowny-Horas R, Retana J (2010) Ant-mediated expansion of an obligate seeder species during the first years after fire. Plant Biol 12: 842-852.

2. Arnan X, Rodrigo A, Retana J (2011) What are the consequences of ant-plant interactions on the abundance of two dry-fruited shrubs in a Mediterranean scrub? Oecologia 167: 1027-1039.

3. Ordoñez JL, Retana J (2004) Early reduction of post-fire recruitment of *Pinus nigra* by post-dispersal seed predation in different time-since-fire habitats. Ecography 27: 449–458.

4. Vander Wall SB, Kuhn KM, Beck MJ (2005) Seed removal, seed predation, and secondary dispersal. Ecology 86: 801–806.
