# Supplementary figures and images for: Uncoupling the Effects of Seed Predation and Seed Dispersal by Granivorous Ants on Plant Population Dynamics
Source: PLoS One. 2012 Aug 7;7(8):e42869. doi: 10.1371/journal.pone.0042869 (PMC3413678; doi:10.1371/journal.pone.0042869)

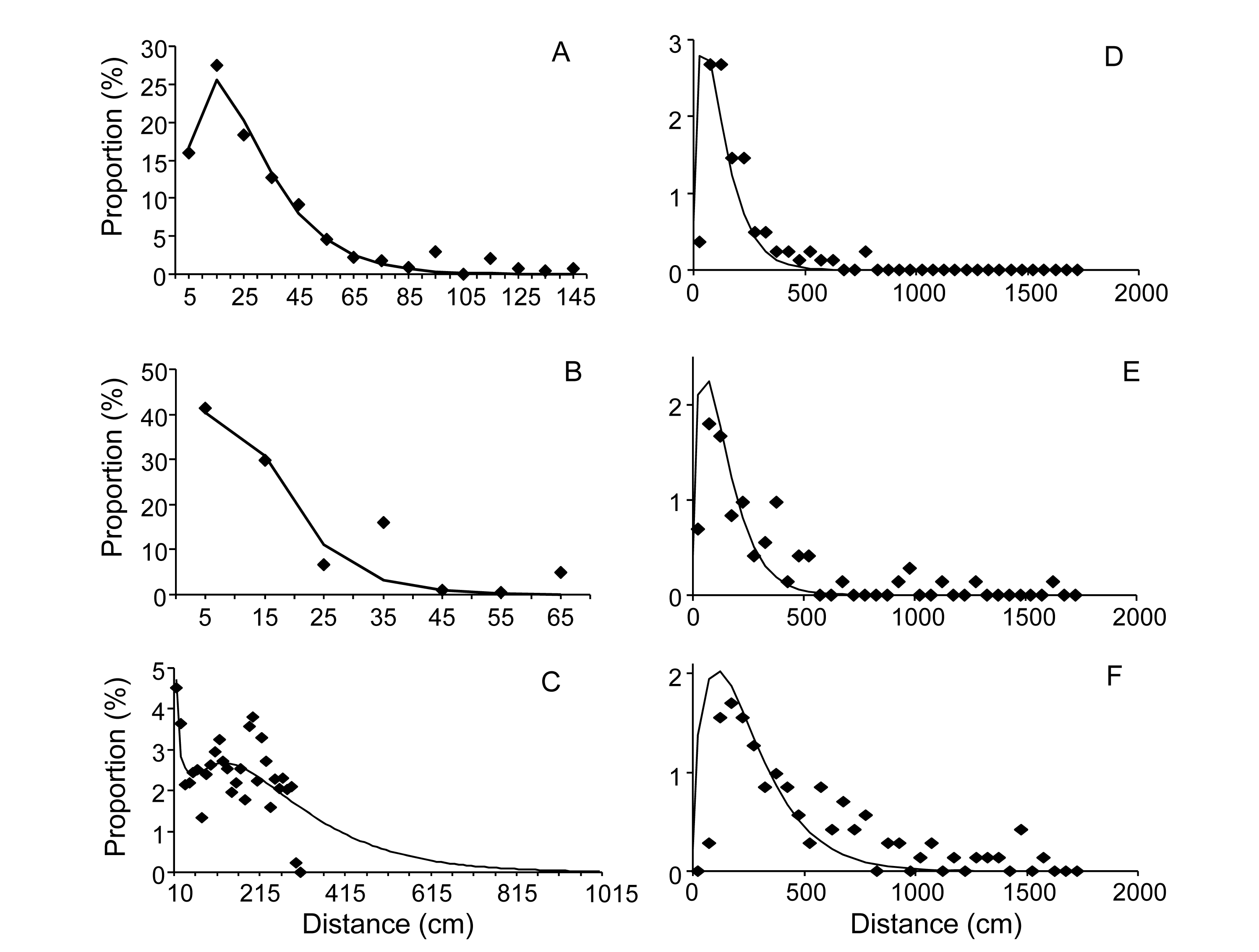

Supplement: Figure S1 — Experimental data and fitted curves for primary and secondary seed dispersal, per plant species. Figures correspond to C. minima (A and D), F. ericoides (B and E) and D. pentaphyllum (C and F). All three figures on the left column show primary dispersal results, whereas those on the right column correspond to seed dispersal by ants through seed drops. (TIF) [file pone.0042869.s002.tif]
