# Supplementary material for: Uncoupling the Effects of Seed Predation and Seed Dispersal by Granivorous Ants on Plant Population Dynamics
Source: PLoS One. 2012 Aug 7;7(8):e42869. doi: 10.1371/journal.pone.0042869 (PMC3413678; doi:10.1371/journal.pone.0042869)
Supplement: Table S1 — Yearly transition matrix (in %) for microhabitat types based on field observations. We first carried out field measurements between 2005 and 2007 and converted those biannual rates to yearly values. Abbreviations: BS, bare soil; LSV, low sparse vegetation; LDV, low dense vegetation; and HV, high vegetation. (DOC) [file pone.0042869.s004.doc]

**Table S1.** **Yearly transition matrix (in %) for microhabitat types based on field observations.**

We first carried out field measurements between 2005 and 2007 and converted those biannual rates to yearly values. Abbreviations: BS, bare soil; LSV, low sparse vegetation; LDV, low dense vegetation; and HV, high vegetation.

|  | BS | LSV | LDV | HV |
| --- | --- | --- | --- | --- |
| BS | 42.9 | 46.9 | 7.3 | 2.9 |
| LSV | 2.0 | 70.7 | 17.3 | 9.9 |
| LDV | 2.0 | 17.0 | 62.0 | 19.0 |
| HV | 0.0 | 4.0 | 4.1 | 91.8 |
