# Supplementary material for: Uncoupling the Effects of Seed Predation and Seed Dispersal by Granivorous Ants on Plant Population Dynamics
Source: PLoS One. 2012 Aug 7;7(8):e42869. doi: 10.1371/journal.pone.0042869 (PMC3413678; doi:10.1371/journal.pone.0042869)
Supplement: Table S2 — Rates (in %) of newly emptied and newly occupied cells as employed in the random mortality module of the simulation algorithm, per plant species. (DOC) [file pone.0042869.s005.doc]

**Table S2.**  **Rates (in %) of newly emptied and newly occupied cells as employed in the random mortality module of the simulation algorithm, per plant species.**

| Plant species | Newly emptied | Newly occupied |
| --- | --- | --- |
| *F. ericoides* | 20.4 | 13.2 |
| *C. minima* | 17.2 | 7.9 |
| *D. pentaphyllum* | 20.9 | 2.1 |
